# Supplementary material for: High-Throughput Volatilome Fingerprint Using PTR–ToF–MS Shows Species-Specific Patterns in Mortierella and Closely Related Genera
Source: J Fungi (Basel). 2021 Jan 19;7(1):66. doi: 10.3390/jof7010066 (PMC7835917; doi:10.3390/jof7010066)
Supplement: Supplementary file 1 [file jof-07-00066-s001.zip › SupplementaryFiles/Supplementary_information.docx]

# Supplementary information

**Mortierellaceae species differ in their produced volatile compounds**

Anusha Telagathoti*, Maraike Probst*, Iuliia Khomenko, Franco Biasioli, Ursula Peintner

SI Fig. 1


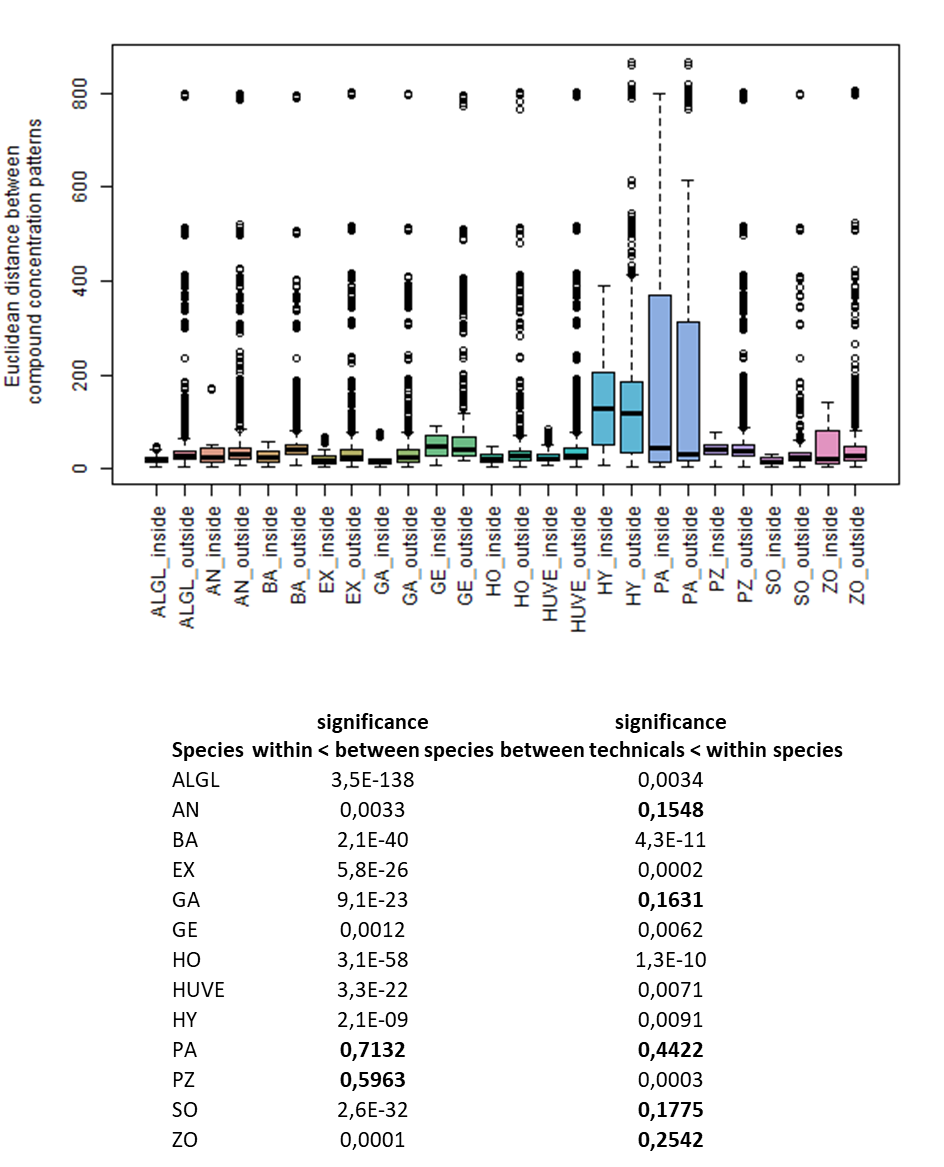


SI Fig. Distances. Boxplot illustrate the pairwise distances between specimens of species. For each species pairwise distances between all technical replicates within a species were collected (<species>_inside; that is distances between biological representatives of a species). For each species, pairwise distances from all technical replications to all replications from all other species were plotted in comparison (<species>_outside; that is distances between species). The middle column of the table illustrates if the distances within a species was smaller compared to the distances to other species. The right column refers to differences only within a species and it indicates if the distances between technical replications of a biological representative were smaller compared to the distances to other biological representatives from the same species (that is, if technical variation on the PTR-TOF was lower compared to biological within species variation). ALGL = *M. alpina/globalpina*, AN = *M. angusta*, BA = *M. bainieri*, EX = *L. exigua*, GA = *L. gamsii*, GE = *M. gemmifera*, HO = *P. horticola*, HUVE = *P. humilis/verticillata*, HY = *L. hyalina*, PA = *E. parvispora*, PZ = *M. pseudozygospora*, SO = *M. solitaria*, ZO = *M. zonata*.
